# Speckle tracking echocardiography data in Brugada syndrome patients

**DOI:** 10.1016/j.dib.2019.104330

**Published:** 2019-07-29

**Authors:** Esther Scheirlynck, Sophie Van Malderen, Andreea Motoc, Øyvind H. Lie, Carlo de Asmundis, Juan Sieira, Gian-Battista Chierchia, Pedro Brugada, Bernard Cosyns, Steven Droogmans

**Affiliations:** a Department of Cardiology, Centrum voor Hart- en Vaatziekten, Universitair Ziekenhuis Brussel, Brussels, Belgium; b Life Sciences and Medicine, Vrije Universiteit Brussel, Brussels, Belgium; c Department of Cardiology, Oslo University Hospital, Rikshospitalet, Oslo, Norway; d Center for Cardiological Innovation, Oslo University Hospital, Rikshospitalet, Oslo, Norway; e Institute for Clinical Medicine, University of Oslo, Oslo, Norway

**Keywords:** Brugada syndrome, Inducible, Mechanical dispersion, Strain, Speckle tracking echocardiography, Spontaneous type 1

## Abstract

Brugada syndrome is characterized by typical electrocardiogram changes and a high risk for sudden cardiac death (Priori et al., 2013). In addition to the well known electrical substrate, morphological and functional alterations appeared to be present in a subset of the Brugada syndrome patients (Catalano et al., 2009). Echocardiographic speckle tracking enables us to detect subtle contraction alterations (Smiseth et al.,2016). We performed transthoracic echocardiography with speckle tracking analysis in 82 healthy controls and 175 Brugada syndrome patients. Main findings are presented and discussed in the article “Contraction alterations in Brugada syndrome; association with life-threatening ventricular arrhythmias” (Scheirlynck et al., 2019). This related Data article contains segmental longitudinal strain values for RV and LV, and the comparison of echocardiographic parameters between Brugada syndrome patients with spontaneous and drug-induced type 1 pattern and between patients with and without ventricular arrhythmia inducibility during electrophysiological study.

**Table:** Specifications Table

Subject	Medicine
Specific subject area	Echocardiography in Brugada syndrome patients
Type of data	Table (3) Supplementary table (1) Figure (2)
How data were acquired	Instruments: Cardiac ultrasound system (Vivid 9; GE Vingmed Ultrasound, Horten, Norway), equipped with a 2D broad-band M3S transducer (2.5 MHz). Offline analysis with EchoPac, version 201 (GE Vingmed Ultrasound, Horten, Norway).
Data format	Raw Analyzed
Parameters for data collection	Brugada syndrome patients (≥18 years old) were recruited from the Brussels University Hospital registry. Exclusion criteria: ischemic or structural heart disease, atrial fibrillation or pacing during echocardiography, insufficient image quality for speckle tracking analysis of both ventricles. Healthy controls with similar age and sex distribution were recruited from the adjacent community.
Description of data collection	Brugada syndrome patients and healthy controls were recruited. Patients either spontaneously presented the diagnostic type 1 electrocardiogram pattern or were diagnosed after drug-challenge. Electrophysiological study was performed to test inducibility of ventricular arrhythmias. In each participant parasternal and apical views were acquired. We measured left and right ventricular dimensions and function. Speckle tracking analysis was performed to obtain left and right ventricular longitudinal strain and mechanical dispersion.
Data source location	Institution: Centrum voor Hart- en Vaatziekten, Universitair Ziekenhuis Brussel City: Brussels Country: Belgium
Data accessibility	With the article
Related research article	Scheirlynck E, Van Malderen S, Motoc A, Lie Ø, de Asmundis C, Sieira J, Chierchia G-B, Brugada P, Cosyns B, Droogmans S Contraction alterations in Brugada syndrome; association with life-threatening ventricular arrhythmias. International Journal of Cardiology 2019 https://doi.org/10.1016/j.ijcard.2019.06.074.

**Table:** 

**Value of the data** • Imaging data in Brugada syndrome patients are scarce. This is one of the largest echocardiographic studies performed on Brugada syndrome patients. • These data can provide insights on structural and functional alterations in Brugada syndrome and drive future research on cardiac imaging in these patients. • The mechanism(s) causing electrical instability in Brugada syndrome are debated. Imaging of the heart in Brugada syndrome might help elucidate pathophysiological mechanisms. • Defining the risk of sudden cardiac death for the individual Brugada syndrome patient is challenging but important for clinical decision making. Echocardiography could improve risk stratification by adding morphofunctional information.

## 1 Data

**Fig. 1:**
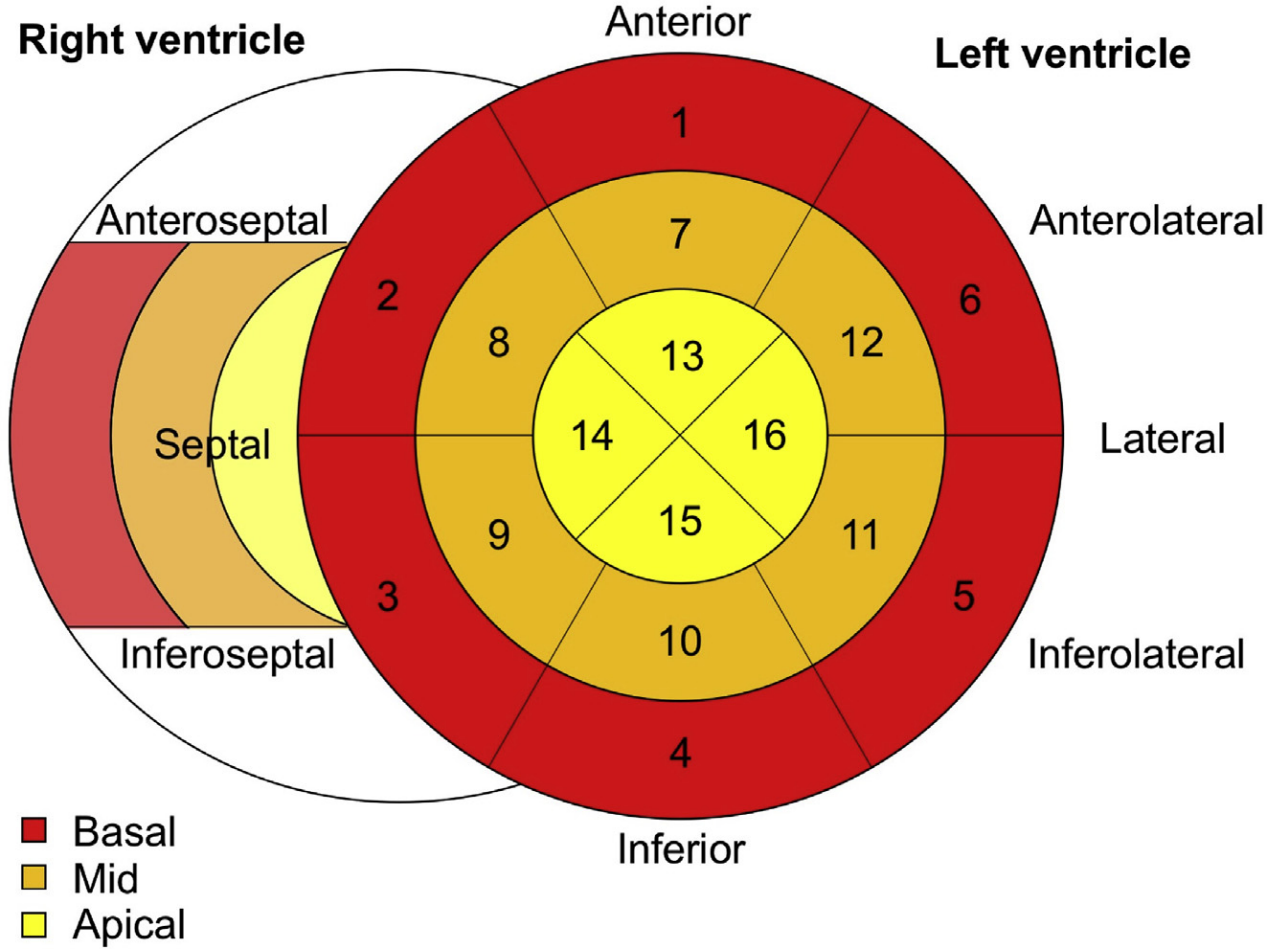
**Speckle tracking segments**. Bullseye plot of the 16 left ventricular and 3 right ventricular free wall segments.

Brugada syndrome is a heritable channelopathy characterized by typical electrocardiogram changes and a high risk for sudden cardiac death [1], [2]. Initially considered as a purely electrical disease, the use of cardiac imaging in Brugada syndrome only gained interest recently. Mild morphological and functional abnormalities affecting the right ventricular outflow tract [3], [4], [5], [6], [7], [8] - and to a lesser extent the right ventricle (RV) and the left ventricle (LV) [9], [10], [11] – were observed. Echocardiographic speckle tracking can reveal subtle alterations in myocardial contraction patterns [12]. We acquired transthoracic echocardiography in 82 healthy controls and 175 Brugada syndrome patients [13]. Speckle tracking analysis was performed for the left (16 segments) and right ventricle (3 segments) (Fig. 1). Segmental longitudinal strain values of healthy individuals and Brugada syndrome patients are reported and compared in Table 1. The raw segmental strain values for Brugada syndrome patients are reported in the supplementary file.

**Table 1:** Segmental longitudinal strain values: BrS patients vs. controls.

	Controls, n = 82	BrS, n = 175	p-value
RVLS apical, % (±SD)	−22.7 (±5.5)	−21.6 (±7.1)	0.25
RVLS mid, % (±SD)	−27.6 (±4.1)	−26.3 (±5.7)	0.04
RVLS basal, % (±SD)	−26.2 (±4.3)	−24.8 (±6.4)	0.06
LVLS anterior basal, % (±SD)	−17.9 (±3.3)	−17.2 (±3.9)	0.18
LVLS anterior mid, % (±SD)	−18.5 (±3.4)	−17.6 (±3.8)	0.08
LVLS anterior apical, % (±SD)	−20.1 (±4.9)	−19.9 (±5.2)	0.75
LVLS anterolateral basal, % (±SD)	−17.4 (±3.3)	−15.7 (±4.8)	0.005
LVLS anterolateral mid, % (±SD)	−17.3 (±3.4)	−16.3 (±3.8)	0.04
LVLS inferolateral basal, % (±SD)	−17.9 (±3.6)	−15.4 (±6.7)	<0.001
LVLS inferolateral mid, % (±SD)	−17.8 (±2.9)	−16.5 (±4.1)	0.02
LVLS lateral apical, % (±SD)	−19.2 (±3.6)	−19.3 (±3.8)	0.87
LVLS inferior basal, % (±SD)	−19.3 (±3.1)	−18.6 (±3.5)	0.16
LVLS inferior mid, % (±SD)	−21.0 (±2.5)	−19.9 (±3.2)	0.004
LVLS inferior apical, % (±SD)	−24.0 (±3.8)	−23.3 (±4.8)	0.22
LVLS inferoseptal basal, % (±SD)	−16.2 (±2.4)	−15.6 (±3.2)	0.09
LVLS inferoseptal mid, % (±SD)	−19.1 (±2.5)	−18.5 (±2.8)	0.11
LVLS anteroseptal basal, % (±SD)	−16.4 (±3.0)	−15.8 (±3.9)	0.15
LVLS anteroseptal mid, % (±SD)	−18.8 (±3.8)	−18.8 (±3.8)	0.94
LVLS septal apical, % (±SD)	−21.4 (±3.9)	−21.7 (±4.1)	0.57

Table 1. Segmental RVLS and LVLS values in healthy individuals and BrS patients. Comparison was performed by Student's t-test.

BrS = Brugada syndrome, LVLS = left ventricular longitudinal strain, RVLS = right ventricular longitudinal strain, SD = standard deviation.

**Fig. 2:**
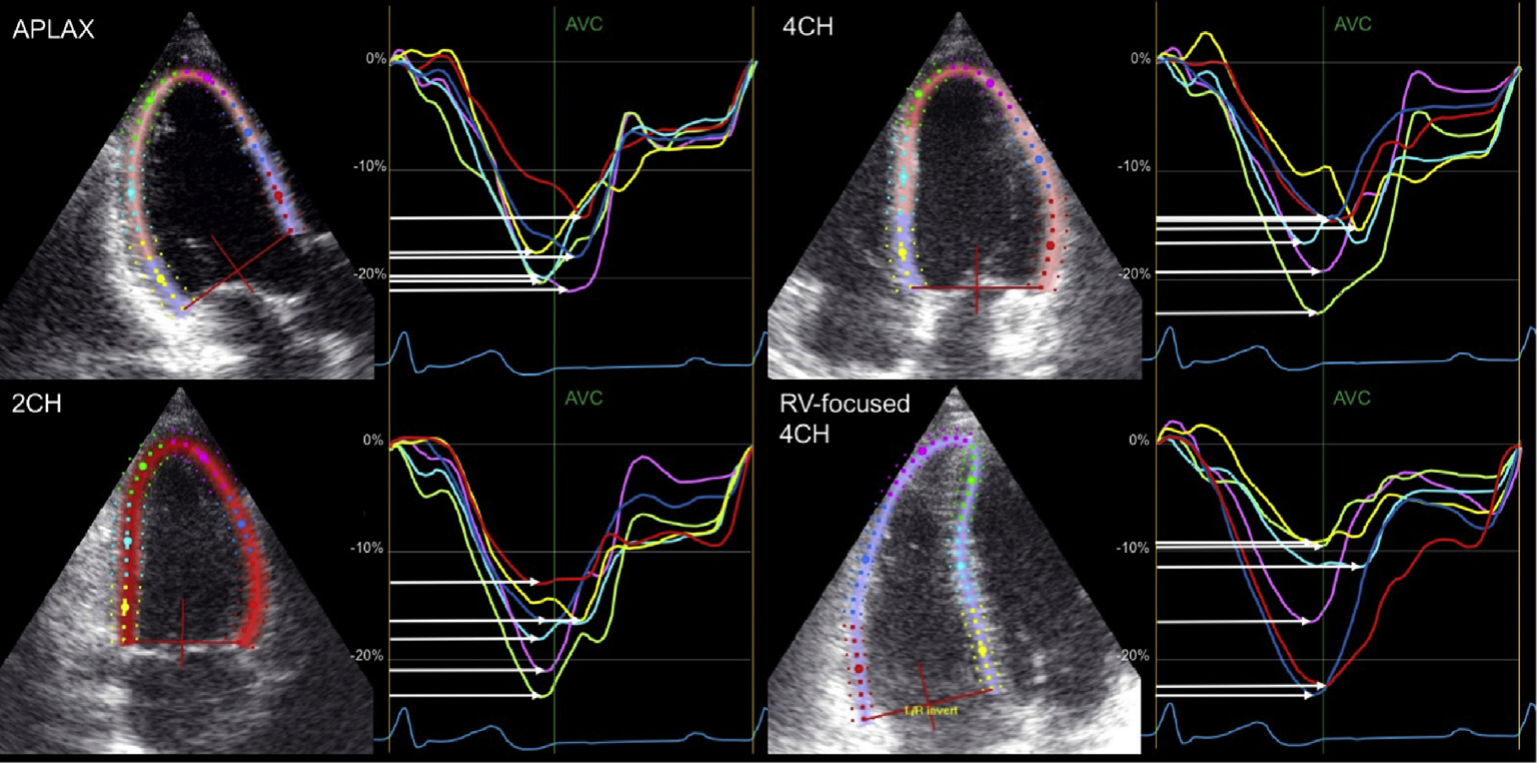
**Mechanical dispersion**. Strain curves of the APLAX, 4-chamber, 2-chamber and RV-focused right ventricle view. The white arrows indicate the time from onset of the QRS complex until time to peak strain in the different segments.

Mechanical dispersion, which is defined as the standard deviation of the time-interval from onset of the QRS-complex until the peak myocardial shortening in the 16 left ventricle, 3 right ventricle free wall and 6 right ventricle segments, was calculated in all patients, as a measure of contraction heterogeneity (Fig. 2).

Echocardiographic data were reported for specific subgroups of Brugada syndrome patients. First, we compared patients who presented a spontaneous type 1 electrocardiogram pattern with patients who were diagnosed based on a drug-induced type 1 pattern (Table 2).

**Table 2:** Demographic and echocardiographic parameters: BrS with drug-induced type 1 vs. BrS with spontaneous type 1.

	BrS with drug-induced type 1 ECG, n = 133	BrS with spontaneous type 1 ECG, n = 42	p-value
**Demographics**			
Age, years (IQR)	47 (33–56)	47 (38–57)	0.32
Women, n (%)	73 (55)	15 (36)	0.03
**TTE**			
LV EF, % (±SD)	58 (±7)	58 (±6)	0.82
LV GLS, % (±SD)	−18.2 (±2.5)	−18.0 (±2.5)	0.74
LV MD, ms (±SD)	36 (±11)	42 (±11)	0.002
RVOTp, mm (IQR)	33 (30–36)	33 (29–36)	0.90
RVOTd, mm (IQR)	21 (18–25)	23 (19–24)	0.30
TAPSE, mm (±SD)	24 (±4)	24 (±7)	0.83
RV FAC, % (IQR)	44 (36–49)	40 (33–46)	0.12
RVLS, % (IQR)	−24.4 (±5.4)	−24.4 (±5.5)	0.95
RV MD, ms (IQR)	21 (13–38)	31 (18–49)	0.04
RVFW MD, ms (IQR)	15 (8–24)	18 (7–37)	0.27

Table 2. Demographic and echocardiographic parameters for LV and RV function. Comparison between BrS patients with drug-induced vs. spontaneous type 1 ECG pattern using Student's t-test, Mann-Whitney U test, or chi-square test as appropriate. BrS=Brugada syndrome, FAC = fractional area change, GLS = global longitudinal strain, LV = left ventricle, LVEF = left ventricle ejection fraction, MD = mechanical dispersion, RV = right ventricle, RVFW = right ventricle free wall, RVLS = right ventricular longitudinal strain, RVOTd = distal right ventricular outflow tract diameter, RVOTp = proximal right ventricular outflow tract diameter, TAPSE = tricuspid annular plane systolic excursion, TTE = transthoracic echocardiography.

Secondly, we compared patients in whom a sustained ventricular arrhythmia could be induced during electrophysiological study compared to patients in whom no ventricular arrhythmia could be induced (Table 3). A total of 168 patients (96%) underwent electrophysiological testing.

**Table 3:** Demographic and echocardiographic parameters: BrS without VT/VF inducibility vs. with VT/VF inducibility at electrophysiological study.

	BrS not inducible, n = 141	BrS inducible, n = 27		p-value
**Demographics**				
Age, years (IQR)	47 (33–56)		47 (38–57)	0.32
Women, n (%)	75 (53)		10 (37)	0.12
**TTE**				
LV EF, % (±SD)	58 (±7)		58 (±7)	0.89
LV GLS, % (±SD)	−18.1 (±2.5)		−18.4 (±2.4)	0.58
LV MD, ms (±SD)	37 (±12)		42 (±10)	0.04
RVOTp, mm (IQR)	33 (29–36)		34 (21–36)	0.54
RVOTd, mm (IQR)	22 (17–24)		22 (19–26)	0.25
TAPSE, mm (±SD)	24 (±5)		25 (±4)	0.32
RV FAC, % (IQR)	42 (35–48)		40 (34–51)	0.88
RVLS, % (IQR)	−24.8 (±5.4)		−23.1 (±5.5)	0.95
RV MD, ms (IQR)	23 (14–41)		22 (17–36)	0.90
RVFW MD, ms (IQR)	15 (8–25)		19 (10–27)	0.34

Table 3. Demographic and echocardiographic parameters for LV and RV function. Comparison between BrS patients without inducibility of VT or VF during electrophysiological testing vs. inducible BrS patients using Student's t-test, Mann-Whitney U test, or chi-square test as appropriate. BrS=Brugada syndrome, FAC = fractional area change, GLS = global longitudinal strain, LV = left ventricle, LVEF = left ventricle ejection fraction, MD = mechanical dispersion, RV = right ventricle, RVFW = right ventricle free wall, RVLS = right ventricular longitudinal strain, RVOTd = distal right ventricular outflow tract diameter, RVOTp = proximal right ventricular outflow tract diameter, TAPSE = tricuspid annular plane systolic excursion, TTE = transthoracic echocardiography, VF = ventricular fibrillation, VT = ventricular tachycardia.

## 2 Experimental design, materials, and methods

### 2.1 Study population

Brugada syndrome patients were recruited from the database of the University Hospital of Brussels. All had been diagnosed with Brugada syndrome based either on a spontaneous or drug induced ST segment elevation with a type 1 morphology of ≥2 mm in 1 or more leads among the right precordial leads (V_1_–V_2_) positioned in the second, third, or fourth intercostal space [1], [2]. Included patients were at least 18 years old. We excluded patients with a history of ischemic or structural heart disease. We also excluded patients with atrial fibrillation or pacing during transthoracic echocardiography (TTE) or with insufficient image quality for speckle tracking analysis of both left and right ventricle, defined as inappropriate tracking of 2 or more segments on a single view.

Healthy controls with similar age and sex distribution were recruited among hospital employees and their family members.

Written informed consent was obtained from all subjects. This study was performed in accordance with the Declaration of Helsinki and was approved by the Ethics Committee of UZ Brussel.

### 2.2 Echocardiography

TTE was performed using a commercial cardiac ultrasound system (Vivid 9; GE Vingmed Ultrasound, Horten, Norway), equipped with a 2D broad-band M3S transducer (2.5 MHz). In each patient standard parasternal long- and short-axis images, apical 4-, 2- and 3-chamber views and a RV focused 4-chamber view were acquired [14]. Lead II was used for ECG recordings. Loops of 3 consecutive heart beats at >50 frames per second were digitally stored for offline analysis (EchoPac, version 201, GE Vingmed Ultrasound, Horten, Norway).

The LV end-diastolic volume (LVEDV) and LV end-systolic volume (LVESV) were measured and the ejection fraction (EF) was calculated by modified Simpson's biplane method. Tricuspid annular plane systolic excursion (TAPSE) was obtained by M-mode through the tricuspid annulus on the 4-chamber view. RV outflow tract (RVOT) proximal and distal diameters were measured from the parasternal short-axis view. End-diastolic and end-systolic RV area (RVA) were obtained from the RV focused 4-chamber view and fractional area change (FAC) was calculated [15].

Speckle tracking analysis was performed on the 3 apical views for the LV and on the RV focused 4-chamber view for the RV [14], [16]. When necessary, the automated region of interest was adjusted to optimise tracking. GLS was defined as the mean of the peak systolic strain in the 16 LV segments and RV longitudinal strain (RVLS) from the 3 RV free wall segments (Fig. 1). The time to peak myocardial longitudinal strain in each segment was measured as the time from onset of the QRS complex on the ECG to maximum myocardial shortening. MD was defined as the standard deviation of the time to peak in respectively the 16 LV segments, the 6 RV segments and the 3 RV free wall segments (Fig. 2) [17], [18].

### 2.3 Drug-challenge

Ajmaline (1 mg/kg) was administered intravenously over a 5-min period to unmask the diagnostic type 1 electrocardiogram pattern in case of non-diagnostic baseline electrocardiogram. The test was considered positive if type 1 ECG ^appeared^ in ≥1 right precordial leads. The drug infusion was discontinued if QRS prolongation exceeded 30%, frequent premature ventricular beats or type 1 Brugada ECG occurred or development of high-degree atrio-ventricular block.

### 2.4 Electrophysiological study

Electrophysiological study protocol consisted of a single site of stimulation at the right ventricular apex, three basic pacing cycles (600, 500, and 430 ms), and introduction of up to 3 ventricular premature beats down to a minimum of 200 ms. A patient was considered inducible if a sustained (≥30 seconds) ventricular fibrillation or ventricular tachycardia was induced.

### 2.5 Statistics

Continuous data was presented as mean ± standard deviation or median with interquartile range (IQR). Categorical data was presented as number (%). Comparisons were performed using T-test, Mann-Whitney U test, χ2 or Fischer's exact test as appropriate.

Statistical analysis was performed using SPSS version 24.0 (SPSS Inc., Chicago, IL, USA).
